# Non-Suicidal Self-Injury in Adolescence: The Role of Pre-Existing Vulnerabilities and COVID-19-Related Stress

**DOI:** 10.1007/s10964-022-01669-3

**Published:** 2022-08-20

**Authors:** Lisa De Luca, Matteo Giletta, Annalaura Nocentini, Ersilia Menesini

**Affiliations:** 1 Department of Education, Languages, Intercultures, Literatures and Psychology, University of Florence, Florence, Italy; 2 Department of Developmental, Personality and Social Psychology, Ghent University, Ghent, Belgium

**Keywords:** Non-suicidal self-injury, COVID-19-related stress, Pre-existing vulnerabilities, Longitudinal study, Adolescence

## Abstract

For many adolescents, the COVID-19 pandemic represents a uniquely challenging period, and concerns have been raised about whether COVID-19-related stress may increase the risk for self-injurious behaviors among adolescents. This study examined the impact of pre-existing vulnerabilities on the occurrence and frequency of Non-Suicidal Self-Injury (NSSI) through COVID-19-related stress, and whether the impact of COVID-19-related stress on NSSI was buffered by the perceived social support during the pandemic. Participants were 1061 adolescents (52.40% females*; M*_*age*_ = 15.49 years, *SD* = 0.76) from a two-wave longitudinal study, which included assessments before the COVID-19 onset and one year later the declaration of the pandemic. Path analyses showed that adolescents with a prior history of NSSI, higher levels of internalizing symptoms, and poor regulatory emotional self-efficacy before the COVID-19 pandemic reported higher levels of COVID-19-related stress which in turn increased their risk to engage in NSSI. Besides, the findings did not support the role of social support as a moderator of the association between COVID-19 related stress and the occurrence/frequency of NSSI. These findings suggest that enhanced stress perception may serve as a key pathway for the continuation and development of NSSI among vulnerable adolescents facing adverse life events.

## Introduction

Adolescence represents a sensitive developmental period characterized by profound biological, cognitive, and social changes, as well as important developmental tasks in the definition of one’s identity and autonomy (Dahl et al., 2018). Adolescence is also a critical period for the onset and development of mental health problems and risky behavior such as Non-Suicidal Self- Injury (NSSI), defined as the direct and deliberate destruction of one’s body tissue without suicidal intent. Given the role of stress in the development of NSSI (Liu and Miller, 2014), the changes in the individual and social environment that occurred during the COVID-19 pandemic might have interfered with adolescent developmental tasks (e.g., Rajkumar, 2020), resulting in the *“perfect storm”* for the emergence and rise of negative outcomes, including NSSI (Branje and Morris, 2021). However, to date, despite the raised concerns about possible increases in self-injurious behaviors across the pandemic (Plener, 2021), little is known about longitudinal changes in NSSI, and about which youth may be at higher risk for NSSI during this period, and why. Indeed, although COVID-19 is a worldwide pandemic, the extent to which it affected youth and therefore, similarly, how it may have influenced NSSI probably depends on prior individual vulnerabilities. This study aimed to examine the extent to which adolescents with pre-existing vulnerabilities, including a prior history of NSSI, higher levels of internalizing symptoms, and poorer regulatory emotional self-efficacy, had a higher risk to engage in NSSI across the pandemic period through higher levels of COVID-19 related perceived stress. Moreover, the extent to which the effects of COVID-19-related perceived stress on NSSI were buffered for adolescents who reported higher levels of social support (i.e., peer and parental support) during the pandemic was investigated.

### COVID-19 Related Stress, Pre-Existing Vulnerabilities, and NSSI

Adverse and negative life events are well-established risk factors for the initiation and maintenance of NSSI, especially when individuals perceive these events as particularly stressful (e.g., Liu et al., 2016). Several theoretical models suggest that individual’s ability to cope with stressful events and to regulate emotions play a critical role to understand the development of psychopathological outcomes in response to stressful situations (e.g., Compas et al., 2017). For youth who face adverse life events and have difficulties to manage negative emotions or to use healthy coping strategies, NSSI may represent a risky coping strategy that serves to regulate their emotions (Gratz and Roemer, 2008). The uncontrollable and unpredictable nature of the COVID-19 pandemic has likely generated elevated and enduring stress for many people with a strong impact not only on public health but also on individuals’ mental health (e.g., Gruber et al., 2020), perhaps especially for some adolescents (Branje and Morris, 2021). Recent work raised concerns about whether COVID-19-related stress may increase the risk for self-injurious behaviors among adolescents. Existing evidence comes primarily from studies that used cross-sectional designs conducted with high school students from Canada (Robillard et al., 2021), Taiwan (Tang et al., 2021), Sweden (Zetterqvist et al., 2021), and with hospitalized adolescents (Du et al., 2021). Only few studies used short-term longitudinal designs with follow-ups conducted only a few months into the COVID-19 pandemic with Chinese children (Zhang et al., 2020) and adolescents (Xiao et al., 2022), and with American high school students (Carosella et al., 2021; Schwartz-Mette et al., 2022). This research revealed high NSSI prevalence around 40.9% in Taiwan (Tang et al., 2021) during the COVID-19 outbreak, and around 42% in China (Zhang et al., 2020) after three months of lockdown. Besides, some of these studies reported an association between COVID-19-related stress and NSSI, suggesting how the pandemic may have led to engage in NSSI (e.g., Xiao et al., 2022). However, no studies evaluated changes in NSSI behavior from before the pandemic to several months into the pandemic, after a prolonged period of stress exposure and uncertainty, which may have further triggered adolescents’ vulnerabilities. Thus, the longer-term impact of the COVID-19 pandemic remains largely unclear, and it is unknown whether some youth may be increased risk for NSSI and why.

According to the diathesis-stress models (van Heeringen, 2012), the psychological stress due to the COVID-19 pandemic may have exacerbated pre-existing psychopathology and developmental vulnerabilities (e.g., Gruber et al., 2020). Recent studies found that adolescents who had specific vulnerabilities before the pandemic, such as higher stress levels (e.g., Branje and Morris, 2021), risky coping (e.g., van Loon et al., 2021), or internalizing problems (e.g., Morales et al., 2022), also experienced more COVID-19 related concerns and perceived stress during the pandemic. Consequently, these adolescents may be also at higher risk for engaging in NSSI. This idea is also consistent with transactional models (Burke et al., 2015) and stress generation models (Hammen, 2006), according to which not only stressful life events may represent risk factors for engaging in NSSI, but also NSSI, as well as internalizing problems, may pose risk for experiencing subsequent elevated stress levels. For example, initial work revealed bidirectional associations between NSSI and interpersonal stress (Miller et al., 2018). Based on this theoretical and empirical work, adolescents with pre-existing vulnerabilities may experience higher levels of stress when exposed to negative life events, such as the COVID-19 pandemic, and consequently they may be at higher risk for engaging in NSSI.

Several intrapersonal factors have been identified as possible vulnerabilities for NSSI engagement. For example, meta-analytic work indicates that a prior history of NSSI is the stronger risk factor for future NSSI engagement (Fox et al., 2015). Furthermore, prior studies found that adolescents with high levels of internalizing problems, such as depressive symptoms (Prinstein et al., 2010), anxiety symptoms (Robinson et al., 2017) and emotion disorders (Bentley et al., 2015), are also at increased risk for subsequent self-injury. These findings are consistent with theoretical models suggesting that NSSI may become a strategy to cope with the internalizing symptoms (Nixon et al., 2002). Thus, a prior history of NSSI and internalizing symptoms could be involved in translating COVID-19-related stress into an increased risk for NSSI.

Difficulties in regulating emotions also represent a crucial factor for understanding why some people engage in NSSI (e.g., Chapman et al., 2006). Adolescents who engage in NSSI experience elevated negative emotions and are less able to regulate them, putting them at increased risk for NSSI. Not only emotion dysregulation but also emotion regulation self-efficacy has been related to NSSI (e.g., Hasking et al., 2018). Emotion regulatory self-efficacy is a cognitive mechanism defined as the belief individuals’ own ability to successfully manage and regulate emotions (e.g., Caprara et al., 2008). Prior work found that higher levels of self-efficacy were associated with lower levels of stress (e.g., Matsushima and Shiomi, 2003). Moreover, self-efficacy is linked to the perception of controllability of a stressful situation, which decreases when individuals’ perception of their own ability to regulate emotions and the consequent management of the event is low (Suldo and Shaffer, 2007). Thus, low confidence in regulating emotions (i.e., regulatory emotional self-efficacy) may result in lower abilities to cope with stressful events, increased perceived stress and in turn higher risk for engaging in NSSI, contributing to the onset and maintenance of this behavior over time (Tatnell et al., 2014). Therefore, adolescents with lower levels of self-efficacy in regulating negative emotions may in turn report a higher level of stress during the COVID-19 pandemic and consequently higher levels of NSSI.

### The Buffering Effect of Social Support

Theoretical and empirical work indicates that individuals who can benefit from positive and supportive interpersonal relationships may be protected from engaging in NSSI. For example, adolescents with higher levels of perceived social support from friends, family, or other significant relationships, report lower levels of NSSI (e.g., Hankin and Abela, 2011). Notably, the protective effects of social support on NSSI may also occur because support buffers the negative impact of stressful events, as suggested by the stress-buffering model (Cohen and Wills, 1985). Prior work found support for the stress-buffering model, underling how a high level of social support may buffer the effects of stress on mental health outcomes and risk behaviors (Rueger et al., 2016), including self-injury (e.g., Liu et al., 2022). Yet, it should be noted that mixed and contrasting evidence also exist (Mackin et al., 2017).

Thus, adolescents who experience higher levels of stress in response to the COVID-19 pandemic, but can benefit from support within their social network, may be less likely to engage in NSSI as compared to their peers with lower levels of support. A recent longitudinal study among female adolescents found that family support served as a protective factor for engaging in NSSI (Carosella et al., 2021), while peer support did not. Specifically, as compared to females who desisted, those who persisted in NSSI engagement from pre- to during the COVID-19 period reported higher levels of perceived stress and lower levels of family support. Building on this work, it is important to understand not only the role of support as protective factor, but also if parental and peer support may buffer the impact of COVID-19-related stress on NSSI behavior.

## Current Study

**Fig. 1:**
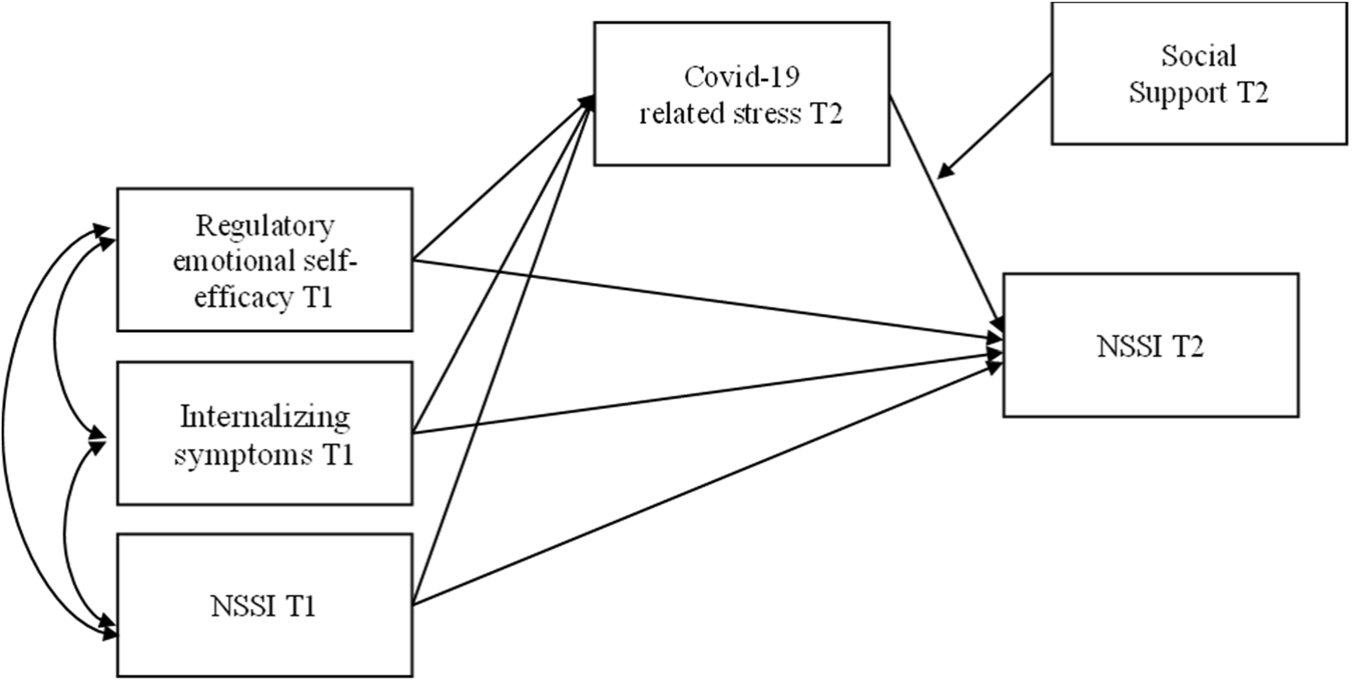
The proposed moderated mediation model

Recent studies suggest that the stress and restrictions due to the COVID-19 pandemic may have resulted in increases in self-injurious behavior among youth. However, research has yet to shed light on the longer-term impact of the pandemic on NSSI, and on which youth may be at higher risk for engaging in NSSI during the pandemic, and why. As such, the present study examines the extent to which adolescents with pre-existing vulnerabilities (i.e., prior history of NSSI, higher levels of internalizing symptoms, and poorer regulatory emotional self-efficacy) reported a higher likelihood to engage in NSSI and a higher NSSI frequency across the pandemic period through higher levels of COVID-19 related stress (see Fig. 1). Moreover, this study aimed to investigate whether the impact of COVID-19 related stress on NSSI was buffered for adolescents who perceived higher levels of social support (i.e., peer support and parental support) during the pandemic. Adolescents with pre-existing vulnerabilities, including a prior history of NSSI, higher levels of internalizing symptoms, and poorer regulatory emotional self-efficacy, may have experienced higher levels of COVID-19-related stress which in turn posed them at higher risk for NSSI (Hypothesis 1). Moreover, higher levels of perceived social support (i.e., peer support and parent support) during the pandemic may have buffered the negative impact of COVID-19-related stress on NSSI (Hypothesis 2).

## Methods

### Participants and Procedure

The sample included 1061 adolescents (52.40% females) enrolled in Grade 9 and 10 of nine high schools in Tuscany (Italy), who participated in at least one of two time points of data collection. The mean age was 15.49 years (*SD* = 0.76) at the baseline, ranging from 14 to 21 years^1^. Most participants were Italians (89.30%), and the remaining adolescents (10.70%) came from different countries. Most participants lived in a two-household family, with both biological parents (84.90%), 14.90% of them lived in a single-parent household, and 0.20% with others from biological parents (e.g., adopted mother).

Participants were part of a longitudinal research project that started in the school year 2019–2020. At baseline, ninth- and tenth-grade students of thirteen high schools were approached to take part in the study (i.e., 70% of total schools contacted). Subsequently, to the school that gave the permission, consent forms were distributed to both students’ families and students themselves to inform them about the project. Only students with parents’ authorization participated in the questionnaire administration. Overall, 91% of the parents provided consent and 85% of the targeted adolescents completed the questionnaire at baseline.

Between January and February of 2020, before the first COVID-19 outbreak in Italy, students in Grade 9 and Grade 10 participated in the baseline assessment (T1; N = 919). Subsequently, participants were assessed again approximately one year later, between December and January of 2020/2021 (T2; N = 579), during the third COVID-19 wave^2^. At Time 1, data collection took place at school under the supervision of a research assistant. At Time 2, the data collection occurred online (i.e., not in presence) due to the COVID-19 restrictions. Research assistants were available online to introduce the survey to the participants and answer any questions.

The retention rate between the two assessments was 47%. Study attrition was mainly due to the decision of some schools not to participate in the follow-up assessment due to the challenges and restrictions related to the COVID-19 situation, which strongly limited data collection conditions. Specifically, four schools and six classes decided not to continue the project for the current school year, which resulted in the loss of 368 participants. Besides, a total of 114 students were not present at T2 due to individual-level factors (e.g., absenteeism). To explore the nature of the dropout at T2, attrition analyses were conducted using multinomial logistic regression models in which dropout at T2 (1 = participation at both waves, 2 = schools’ dropout due to COVID-19 restrictions, 3 = individual dropout - e.g., absenteeism) was predicted separately by the main study variables at T1. Results showed that, as compared to students who participated at both time points, those who did not participate at T2 because of school dropout reported higher levels of prior NSSI (OR = 1.667, 95% CI [1.251, 2.223], *p* < 0.001) and internalizing symptoms (OR = 2.017, 95% CI [1.502, 2.708], *p* < 0.001), as well as poorer regulatory emotional self-efficacy (OR = 1.487, 95% CI [1.251–1.767], *p* < 0.001). However, no differences were found between participants who were present at both time points and those who did not complete the questionnaire at T2 due to individual-level factors (i.e., NSSI: OR = 1.289, 95% CI [0.840, 1.977], *p* = 0.245; internalizing symptoms: OR = 1.332, 95% CI [0.861, 2.061], *p* = 0.199; regulatory emotional self-efficacy: OR = 0.995, 95% CI [0.775, 1.278], *p* = 0.968). Thus, all students with data on at least one time point were included in the main analyses (N = 1061).

Moreover, 142 adolescents took part in the data collection only at Time 2, due to individual-level factors as absenteeism during the first data collection. To compare participants with and without missing data, a Little’s (1988) Missing Completely at Random (MCAR) test was performed. Although the test emerged to be significant, χ^2^ (22) = 38.302, *p* = 0.017, the normed χ^2^/df of 1.740 suggested that data were likely missing at random supporting the inclusion of participants with missing data in the analyses (Bollen, 1989). Missing data were handled using the Full Information Maximum Likelihood estimation (FIML, Acock, 2005) that allows retaining cases with missing data, therefore avoiding potentially biased parameter estimates through pairwise or listwise deletion (Schafer and Graham, 2002). The study received ethical approval from the University’s Committees for Research.

### Measures

#### Internalizing symptoms

Internalizing symptoms were assessed at T1 using nine items from the sub-scale anxious and depressive symptoms of the Youth Self-Report (YSR; Achenbach et al., 2001). Participants were asked to rate each item (e.g., “I am nervous or tense”, “I am too fearful or anxious” and “I am unhappy, sad, or depressed”) on a three-point Likert scale, from 0 (“*not true*”*)* to 2 (‘*somewhat or sometimes true*), referring to the past 6 months. For each participant, answers to the items were averaged (Cronbach’s α = 0.85) with higher scores indicating higher levels of anxious/depressive symptoms.

#### Poor regulatory emotional self-efficacy

Poor regulatory emotional self-efficacy was assessed at T1 using four items from the Perceived Emotional Self-Efficacy Scale (Caprara and Gerbino 2001). This self-report measure includes three subscales tapping into the self-efficacy construct (i.e., self-efficacy in the expression of positive emotions, self-efficacy in the management of negative emotions, and empathetic self-efficacy). In this study, only the negative emotions subscale was used (e.g., “overcome the frustration if others don’t appreciate you as you would like”; “keep yourself calm in stressful situations”). Participants rated each item on a five-point Likert scale ranging from 1 (“not all capable”) to 5 (“entirely capable”) and subsequently their answers were reverse-scored, so that higher values indicate poorer levels of regulatory emotional self-efficacy. A total score was computed by averaging across the four items (Cronbach’s α = 0.72).

#### COVID-19-related perceived stress

COVID-19-related perceived stress was measured at T2 using the Perceived Stress Scale (PSS-10; Cohen et al., 1983; Mondo et al., 2019). This scale includes ten items rated on a 5-point Likert scale from 1 (‘*Never*’) to 5 (‘*Very often’*); six items are negatively stated (e.g., “in the last month, how often have you felt angered because of things that were outside your control?”) and four are positively stated (e.g., “in the last month, how often have you felt confident about your ability to handle your personal problems”). The scale was preceded by a brief text explaining what a stressful event is and that the health emergency due to COVID-19 can be defined as a stressful event. Thus, the students were asked to respond to the items on how they felt in the last month, referring to COVID-19 (e.g., Morales et al., 2022). After reverse scoring answers to the positive items, a total PSS-10 score was computed by averaging across the ten items (Cronbach’s α = 0.87). In the present sample, CFAs showed good fit for the assessment of perceived stress (χ^2^(34) = 140.923, *p* < 0.001 CFI = 0.953; TLI = 0.938, RMSEA = 0.074, 90% CI [0.061, 0.087]).

#### Perceived social support

Perceived social support was measured at both time points using the Multidimensional Scale of Perceived Social Support (MSPSS; Zimet et al., 1988). This scale includes three subscales to assess perceived social support by the family (e.g., “my family tries to help me; I can talk about my problems with my family”), by peers (e.g., “I can count on my friends when things go wrong”; “I have friends with whom I share joys and sorrows”) and by a significant other (e.g., “There is a special person around when things go wrong”; “there is a special person who cares about feelings”). Each subscale includes four items rated on a seven-point Likert scale ranging from 1 *(‘very strongly disagree’*) to 7 (‘*very strongly agree*’). In this study, the “significant other” subscale was not included. Total scores of family support and peer support were computed by averaging items with higher scores indicating higher levels of perceived social support for each dimension (Cronbach’s α = 0.92 and 0.91 for peer support and parental support, respectively).

#### Non-suicidal self-injury

Non-Suicidal Self-Injury (NSSI) was measured at both time points using six items that assessed different types of NSSI behavior (e.g., cutting/carving, burning, hitting, scraping/picking skin to the point of bleeding, biting, inserting objects under the skin/nails; Prinstein, 2008). Participants were asked to indicate on a five-point Likert scale from “never” to “10+times” how many times in the previous year they intentionally engaged in each of these behaviors, without suicidal intent. A total score of NSSI was computed averaging across participants’ answers to the six items, with higher scores indicating higher levels of NSSI engagement (Cronbach’s α = 0.83 and 0.85 at T1 and T2 respectively). Subsequently, the NSSI variable at T1 was dichotomized, distinguishing between adolescents who engaged in any of the NSSI behaviors at least once (i.e., Yes) and adolescents who did not report engaging in NSSI (i.e., No). Instead, NSSI at T2 was used as an average value and then modeled with the two-part model.

### Analysis Plan

Analyses were carried out consistent with the preregistration (see https://osf.io/xa6vm/?view_only=58b2eec0376b483ba25abf2239f2ec26), unless differently indicated. First, descriptive analyses, including paired t-tests and bivariate correlations, were computed to examine changes in NSSI over time—among participants who completed both assessments—and associations among all study variables.

Second, path analyses were used to test the direct and indirect effects of pre-existing vulnerabilities at T1 on the occurrence and frequency of NSSI at T2, throughout COVID-19-related perceived stress at T2 (see Fig. 1). Subsequently, interaction effects between social support (i.e., peer support and parental support) and COVID-19 related stress on NSSI at T2 were tested. Notably, two separate models were estimated to examine the moderating effects of peer and parental support, respectively. Significant direct and indirect effects were evaluated based on the associated 95% confidence intervals, from *k* = 1000 bootstrap re-samples, not containing zero (Hayes and Scharkow, 2013).

To model NSSI at T2 and deal with the non-normal distribution of NSSI, two-part models were used. Two-part models are often used to model variables with a large number of zero values (i.e., floor effects) and they allow the simultaneous prediction of both the likelihood of a certain behavior to occur (e.g., NSSI) as well as the frequency of the behavior, among those who report it. In the two-part model, continuous data can be treated as a mixture of zero values (i.e., responses that assume a value of zero) and continuous values (i.e., other responses that have a continuous distribution; Olsen and Schafer, 2001). Thus, given the use of two-part models, all analyses included two different outcomes, that is, the occurrence (i.e., yes/no) and the frequency (i.e., continuous values) of NSSI at T2.

Several additional analyses were also conducted. As exploratory analyses, the role of gender as a moderator of the relationship between pre-existing vulnerabilities and NSSI at T2, through COVID-19-related stress was examined. Gender moderation was tested with a multi-group approach, comparing a freely estimated model (e.g., without constraints across the two different groups) with a constrained model in which the different paths were fixed to be equal across gender. Moreover, the possibility that changes in social support from pre- to post-pandemic may have been more relevant to reduce the effect of COVID-19-related stress on NSSI was explored. Indeed, for several adolescents perceived social support may have decreased during the pandemic, and therefore only adolescents with stable high or increasing social support may have been buffered. Thus, a three-way interaction between social support at T1, T2, and COVID-19-related stress on NSSI at T2 was added to the model. Sensitivity analyses were also carried out. First, the main models were examined only among adolescents whose schools participated in both waves of data collection (*N* = 693) to test the robustness of findings, given the high drop-out. Second, the main analyses were conducted with the NSSI variables calculated only based on the more severe four items (i.e., cutting/carving, burning, scrapping, inserting object under the skin/nails) instead of the original six items. These latter analyses were performed to test if findings were consistent when excluding mild forms of NSSI (i.e., self-biting, self-hitting). Thus, the NSSI prevalence has been re-calculated considering only the more severe forms (four items) and removing the mild forms of NSSI (two items).

## Results

### Descriptive Analyses

Table 1 reports bivariate correlations, means, and standard deviations for all study variables. Among adolescents who participated at both time points (*N* = 437), 33.10% reported NSSI at Time 1 and 34.80% at T2. Moreover, among these adolescents the frequency of NSSI did not differ significantly at the two assessments, *M* = 1.21, *SD* = 0.44 at T1 and M = 1.24, *SD* = 0.51 at T2, *t* (433) = −1.892, *p* = 0.059, suggesting the lack of an average increase in NSSI from before to the COVID-19 pandemic assessment.

**Table 1:** Bivariate correlations among the study variables

	1	2	3	4	5	6	7
1. Internalizing symptoms T1	1						
2. Poor regulatory emotional self-efficacy T1	0.52^**^	1					
3. COVID-19 related stress T2	0.58^**^	0.48^**^	1				
4. Peer support T2	−0.29^**^	−0.15^**^	−0.31^**^	1			
5. Parental support T2	−0.36^**^	−0.22^**^	−0.51^**^	0.43^**^	1		
6. NSSI T1	0.40^**^	0.25^**^	0.37^**^	−0.24^**^	−0.39^**^	1	
7. NSSI T2	0.35^**^	0.16^**^	0.43^**^	−0.40^**^	−0.44^**^	0.46^**^	1
Mean	0.73	2.99	1.99	5.56	5.65	38.70%	0.08
Sd	0.48	0.83	0.73	1.31	1.37	–	0.14

NSSI at T1 was dichotomized; thus we report the proportion of adolescents engaging in NSSI. For NSSI at T2 log_10_ values are reported

*NSSI* non-suicidal self-injury

**p < 0.01

### Pre-Existing Vulnerabilities, COVID-19 Related Stress, and NSSI

As a first step, the direct path from pre-existing vulnerabilities to NSSI without the indirect effect of COVID-19-related stress were examined. Regarding the occurrence of NSSI, results showed a significant positive effect from the prior history of NSSI (β = 0.446, *SE* = 0.050, *p* < 0.001) and internalizing symptoms (β = 0.175, *SE* = 0.061, *p* = 0.004) on NSSI at T2. No significant effect of poor regulatory emotion self-efficacy on NSSI at T2 was found (β = −0.066, *SE* = 0.067, *p* = 0.327). Regarding the frequency of NSSI, results showed only a significant positive effect of internalizing symptoms on the frequency of NSSI at T2 (β = 0.318, *SE* = 0.101, *p* = 0.002). No significant effects were found from the prior history of NSSI (β = 0.140, *SE* = 0.096, *p* = 0.147), and regulatory emotion self-efficacy on the frequency of NSSI at T2 (β = −0.051, *SE* = 0.096, *p* = 0.599).

**Fig. 2:**
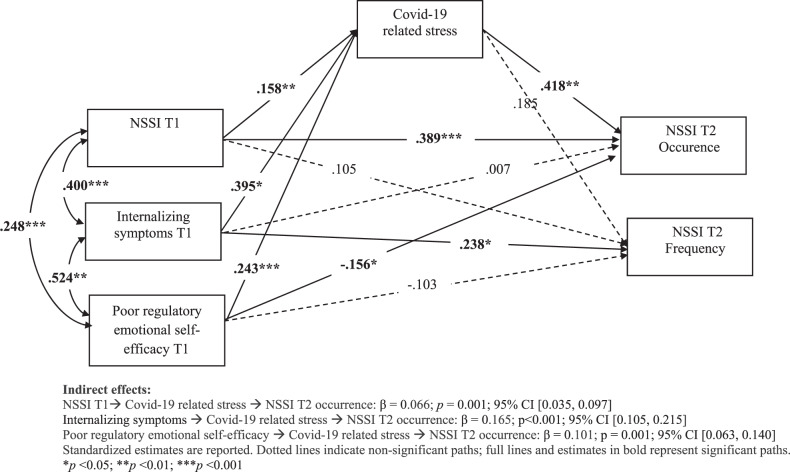
Mediation model predicting NSSI at T2 from pre-existing vulnerabilities via COVID-19-related stress

Figure 2 displays the direct and indirect effects of the final model, including COVID-19-related stress; model estimates for all paths are reported in Table 2. The findings showed significant positive effects of a previous history of NSSI, internalizing symptoms, and poor regulatory emotional self-efficacy on COVID-19-related stress, which in turn was positively associated with NSSI occurrence, but not frequency, at T2 (see Table 2). The indirect effect of prior history of NSSI, internalizing symptoms, and poor regulatory emotional self-efficacy on the occurrence of NSSI at T2 through COVID-19-related stress were all significant (see Fig. 2). Thus, adolescents with pre-existing vulnerabilities perceived the COVID-19 period as more stressful, and this in turn led to a higher likelihood to engage in NSSI. No significant indirect effects were found on the frequency of NSSI.

**Table 2:** Estimates from path model predicting NSSI occurrence and frequency at T2 by pre-existing vulnerabilities via COVID-19-related stress

Outcome	Predictors	β	SE	95% C.I.	*P* value	*R*²	OR	95% C.I.
NSSI T2 occurence	NSSI T1	0.389***	0.047	0.311–0.467	<0.001	0.380	6.319**	4.051–9.856
	Internalizing symptoms T1	0.007	0.066	−0.102–0.115	0.921		1.032	0.610–1.744
	Poor regulatory emotional self-efficacy T1	−0.156*	0.065	−0.264–0.049	0.017		0.648**	0.476–0.883
	COVID-19 stress T2	0.418***	0.063	0.314–0.521	<0.001		3.750**	2.558–5.497
NSSI T2 frequency	NSSI T1	0.105	0.101	−0.062–0.271	0.300	0.150	–	–
	Internalizing symptoms T1	0.238*	0.112	0.054–0.423	0.034		–	–
	Poor regulatory emotional self-efficacy T1	−0.103	0.099	−0.267–0.060	0.298		–	–
	COVID-19 stress	0.185	0.107	0.009–0.361	0.083		–	–
COVID-19 stress T2	NSSI T1	0.158***	0.041	0.091–0.226	<0.001	0.410	–	–
	Internalizing symptoms T1	0.395***	0.040	0.329–0.462	<0.001		–	–
	Poor regulatory emotional self-efficacy T1	0.243***	0.052	0.158–0.327	<0.001		–	–

*NSSI* non-suicidal self-injury

*p < 0.05, **p < 0.01, ***p < 0.001

### The Moderating Role of Social Support

In the model examining the moderating effect of perceived peer support on the association between COVID-19-related stress and NSSI, no significant interaction effects were found both on the occurrence of NSSI (β = −0.010, *SE* = 0.059, *p* = 0.870) and on the frequency of NSSI at T2 (β = −0.054, *SE* = 0.066, *p* = 0.416). Similar results were also observed with respect to the interaction effects between perceived parental support and COVID-19-related stress on the occurrence as well as on the frequency of NSSI at T2 (β = −0.055, *SE* = 0.065, *p* = 0.396 and β = 0.025, *SE* = 0.080, *p* = 0.754, respectively). Thus, the associations between COVID-19-related stress and NSSI did not differ across adolescents experiencing different levels of social support. Given the absence of significant interactions, moderated mediation models were not examined further. However, the results highlight a significant and negative direct effect of perceived parental support on the occurrence (β = −0.172, *SE* = 0.065, *p* = 0.008) and on the frequency (β = −0.221, *SE* = 0.080, *p* = 0.006) of NSSI at T2. No significant associations were found for perceived peer support.

### Supplementary Analyses

Exploratory analyses were conducted to examine gender differences and the possible moderating role of changes in social support over time. Multi-group analyses revealed that constraining all paths to be equal across gender did not worsen the model fit, indicating that the effects of pre-existing vulnerabilities on NSSI via COVID-19-related stress were similar for boys and girls, Δχ^2^ (14) = 7.430, *p* = 0.917. Moreover, the three-way interactions between social support at T1, T2, and COVID-19-related stress (see Supplemental Material available online) did not show a significant effect both on the presence and frequency of NSSI at T2.

Sensitivity analyses including only schools that participated in both time points of data collection yielded results consistent with the ones emerged in the whole sample, confirming the indirect effects of pre-existing vulnerabilities on the occurrence of NSSI through COVID-19 related stress (indirect effects: prior history of NSSI: β = 0.065, *SE* = 0.020, *p* = 0.001; internalizing symptoms: β = 0.164, *SE* = 0.028, *p* < 0.001; poor regulatory negative emotional self-efficacy: β = 0.104, *SE* = 0.030, *p* < 0.001). Similarly, results focusing on the more severe forms of NSSI revealed indirect effects of pre-existing vulnerabilities on the occurrence of NSSI through COVID-19 related stress (indirect effects: prior history of NSSI: β = 0.038, *SE* = 0.015, *p* = 0.011; internalizing symptoms: β = 0.132, *SE* = 0.031, *p* < 0.001; poor regulatory negative emotional self-efficacy: β = 0.076, *SE* = 0.027, *p* = 0.004). For additional details see sensitivity analyses in the Supplemental Material available online.

## Discussion

Numerous concerns have been raised about the possible impact of the COVID-19 pandemic on adolescent NSSI (e.g., Plener, 2021). However, because previous studies have used cross-sectional (e.g., Tang et al., 2021) or short-term longitudinal designs (e.g., Xiao et al., 2022), little is known about the longer-term changes in NSSI across the pandemic and about *who* (e.g., which youth) may be at higher risk for NSSI during this period and *why*. This study addressed these gaps by examining whether, during the first year of the pandemic, adolescents with pre-existing vulnerabilities were more likely to engage in NSSI, through higher levels of COVID-19-related stress.

This study’s findings suggest an indirect effect of pre-existing vulnerabilities on NSSI through higher levels of COVID-19-related stress. First, it is important to note that in contrast with concerns raised about an increase in NSSI during the pandemic (e.g., Robillard et al., 2021), the current study found no evidence that the prevalence or average frequency of NSSI increased during the pandemic. Instead, only adolescents with pre-existing vulnerabilities emerged to be at higher risk for NSSI during the pandemic. Notably, prior studies evaluating the impact of the pandemic on adolescents’ mental health showed high heterogeneity and mixed results (e.g., Branje and Morris, 2021). The current study stresses this point, showing that the pandemic was perceived as more stressful by adolescents who were already vulnerable, and this may have contributed to their increased likelihood of NSSI engagement. Specifically, adolescents with a prior history of NSSI, higher levels of internalizing symptoms, and poorer regulatory emotional self-efficacy experienced higher levels of COVID-19-related stress, which in turn was associated with a higher likelihood to engage in NSSI (i.e., occurrence). The findings are consistent with previous work, according to which vulnerable adolescents are at higher risk to experience stress, especially during emotionally challenging periods such as the COVID-19 pandemic (Guessoum et al., 2020). In fact, previous studies revealed that youth who reported higher stress levels, more risky coping strategies and more internalizing problems also experienced more stress during the pandemic (van Loon et al., 2021). In a sensitive period of transition such as adolescence, the changes in the individual and social environment due to the COVID-19 restrictions, for example, social distancing, the interruption, and drastic changes of in-person learning, daily activities, and the deprivation of school and extra-familial support may have increased social isolation and sense of loneliness, contributing to exacerbate vulnerabilities, and leading to experience higher levels of psychological distress (e.g., Breaux et al., 2021). Given the role that stress plays in NSSI engagement, the COVID-19 pandemic may have represented a particular concern for the development and maintenance of this behavior (Carosella et al., 2021), especially for vulnerable adolescents. A strong experience of stress, such as COVID-19-related stress, could have led to difficulties to deal with intense and uncontrollable emotions, including anger, frustration and sadness (Stänicke et al., 2019). Consequently, consistent with theoretical and empirical research on the functions of NSSI (Chapman et al., 2006), the avoidance of negative emotions due, for example, to stressful events may have had a central role in explaining NSSI engagement among vulnerable adolescents. In fact, NSSI may provide immediate relief from emotional distress in a specific moment (Armey et al., 2011), representing a risky coping strategy, for example to down-regulate arising negative feelings (e.g., Liu et al., 2016).

Differences were found in the prediction of NSSI occurrence versus frequency. First, the indirect effects were only observed for NSSI occurrence, but not frequency, suggesting that adolescents with pre-existing vulnerabilities perceived higher levels of COVID-19-related stress which increased their risk to engage in NSSI (i.e., NSSI occurrence), but not to engage in it more frequently (i.e., NSSI frequency). This may indicate that adolescents who already engaged in NSSI before the pandemic did not necessarily show a higher NSSI frequency (e.g., severity) during the pandemic. These findings could suggest how vulnerable adolescents, during a stressful period as COVID-19, may have engaged in NSSI as a temporary strategy to regulate their emotions, therefore explaining the increasing of the occurrence. In fact, stressful situations are often proximal triggers for the engagement in NSSI (e.g., Liu et al., 2016), and consequently, NSSI may represent a risky coping strategy to regulate stressful event in the short run (e.g., Nixon et al., 2002). Second, results showed that only internalizing symptoms predicted higher levels of NSSI (e.g., frequency) from pre- to post-pandemic, suggesting how the presence of these symptoms lead directly to a higher frequency of NSSI behavior regardless of the perception of stress-related to COVID-19. These findings are consistent with prior work, indicating that adolescents with depressive symptoms tend to engage more frequently in NSSI behaviors (Valencia-Agudo et al., 2018).

Finally, the study extended prior findings related to the role of social support in the engagement of NSSI during the pandemic (Carosella et al., 2021), exploring the role of peer and parental support in buffering the impact of COVID-19-related stress on NSSI. In contrast to stress-buffering theories (Cohen and Wills, 1985), findings did not support the role of social support as moderator of the association between COVID-19-related stress and the occurrence/frequency of NSSI. Irrespective of the amount of support adolescents perceived, COVID-19-related stress posed them at higher risk for NSSI. These findings suggested that also parental support did not buffer the effect of perceived stress on the engagement in NSSI, probably for the poor or negative quality of parental support during the confinement. In fact, even if in some cases the functioning of the family may not have been affected by the pandemic, on the other hand some families may have been severely affected by the stress of the pandemic (e.g., Fontanesi et al., 2020), probably aggravating existing vulnerabilities. However, parental support, but not peer support, was directly associated with lower levels of NSSI engagement. This finding is consistent with prior work that revealed a protective role of family, but not of peer support on NSSI during the pandemic period (Carosella et al., 2021). Despite existing evidence supporting the stress-buffering hypothesis, several studies also found no support for it (Mackin et al., 2017). Consistent with some of these studies, data seem to support a main-effect model (Rueger et al., 2016) rather than a stress-buffering model, according to which support may have a direct impact on well-being, independently of stress exposure.

This study has several strengths, including the large sample and the two-wave design with data collected before and during the COVID-19 pandemic, which allowed us to examine possible changes in NSSI as a function of COVID-19-related stress. Moreover, the use of the two-part model allowed us to examine the effects of pre-existing vulnerabilities and the COVID-19-related stress both on the occurrence and frequency of NSSI.

Despite these strengths, the study’s findings should be interpreted considering several limitations. First, the self-report assessment of NSSI may have been affected by social desirability, respondent, and recall bias, leading to possible misinterpretation in the definition of NSSI. To address this limitation, an integrated methodology that also captures the qualitative dimension in addition to the quantitative dimension, such as interviews and focus groups, could be useful. Second, at both time points NSSI engagement in the past year was assessed, whereas, for some adolescents, only 10/11 months have passed between the first (T1) and second (T2) data collection. Third, restrictions related to the COVID-19 emergency (e.g., distance learning) limited data collection, leading some schools to decline participation to the second administration of the questionnaire and therefore to a substantial loss of participants across the two waves. Besides, the change in the survey method between the two waves of data collection (i.e., in presence vs remote) may have influenced participants’ responses, especially for a stigmatized behavior such as NSSI, as some youth may have been more willing to report engaging in NSSI in a certain situation. Fourth, the study is culture-specific, and the findings cannot be generalized to other cultures. Relatedly, participants’ socio-demographic information, such as culture/geographic background, income, education, and socioeconomic status (SES), were not assessed. Fifth, the study included only two-time points when ideally three waves of data are needed to examine mediation effects. Finally, it is important to keep in mind that the pandemic has gone through different phases, and it involved several changes and transitions that have reflected both on the individual and on the living environment. Thus, to examine the longer-term impact of the COVID-19 pandemic, a design that combines intense and shorter-term assessments (e.g., daily assessments) with longitudinal assessments over the course of several months or one year would be more suitable to better understand changes over time (see Schwartz-Mette et al., 2022).

The COVID-19 pandemic offered a unique natural context to examine the impact of adversity on NSSI; yet the implications of this work extend beyond this unique event. Indeed, the study’s findings contribute to a better understanding of how other potentially traumatic events may increase the risk for the development and maintenance of NSSI among vulnerable youth. In this regard the findings suggest that in a sensitive period of transition such as adolescence, the changes in the individual and social environment due to the COVID-19 restrictions might have interfered with the successful achievement of adolescents’ developmental tasks, contributing to rise mental health problems (e.g., Rajkumar, 2020). Practical implications could involve the importance of focusing on regulatory emotional self-efficacy as a protective factor. In fact, the ability to manage emotions could have a crucial role in preventing individual’s adjustment problems, such as self-injury behavior, and to deal with stressful events like COVID-19 pandemic. Finally, along with previous discussion, since NSSI may be used as an unhealthy coping strategy, these results highlight the importance of teaching strategies, through tailored training programs aimed to adolescents with certain vulnerabilities, to deal with adverse life events.

## Conclusion

The tumultuous months of the COVID-19 pandemic, characterized by high levels of uncontrollability, unpredictability, and social restrictions, have raised serious concerns about the possibility that among adolescents, who show enhanced sensitivity to stress, rates of self-injurious behaviors may have peaked. However, prior evidence is limited, and it remains unknow whether some adolescents more than others were at risk for NSSI throughout the pandemic. This study contributed to address this research gap revealing that NSSI rates did not increase during the COVID-19 pandemic; yet adolescents with pre-existing vulnerabilities were at higher risk for distress during the pandemic, leading to a greater probability to engage in NSSI. Furthermore, the findings highlight that both peer and parental support did not buffer the effect of COVID-19-related stress on the occurrence/frequency of NSSI. These findings can help us begin to better understand which adolescents were more at-risk for engaging in self-injurious behavior, especially during negative and uncontrollable life events such as the COVID-19 pandemic.

## Preregistration

All study hypotheses, as well as study design, the analytic approach, and secondary analyses, were preregistered in Open Science Framework (OSF) (see https://osf.io/xa6vm/?view_only=58b2eec0376 b483ba25abf2 239f2ec26).

## Supplementary information

Supplementary Information

## Footnotes

1 The frequency of the age was as follow: 21 (2%) had 14 years old; 623 (58.80%) had 15 years old; 313 (29.60%) 16 years old; 86 (8.20%) 17 years old; 14 (1.30%) 18 years old; 2 (0.20%) 19 years old and 1(0.10%) 21 years old.

2 The second survey took place almost a year after the first when new restrictive and containment measures were introduced. With the DPCM of November 3, 2020, in consideration of the particularly widespread nature of the pandemic and the increase of COVID-19 cases on the national territory, new provisions limiting the teaching activities in presence were progressively introduced. Specifically, the use of distance learning for high schools was introduced. In fact, during the survey, most of the schools that participated in the project were in distance learning, while others were in mixed mode teaching.
