# Supplementary material for: Non-Suicidal Self-Injury in Adolescence: The Role of Pre-Existing Vulnerabilities and COVID-19-Related Stress
Source: J Youth Adolesc. 2022 Aug 20;51(12):2383–95. doi: 10.1007/s10964-022-01669-3 (PMC9392436; doi:10.1007/s10964-022-01669-3)
Supplement: Supplementary file 1 — Supplementary Information [file 10964_2022_1669_MOESM1_ESM.docx]

NSSI AND COVID-19 IN ADOLESCENCE

Supporting information for **Non-Suicidal Self-Injury in Adolescence: The Role of Pre-existing Vulnerabilities and Covid-19 Related Stress**

- Additional Information on Sensitivity Analysis – Mediation Model with Schools that Participated at both Waves
- Additional Information on Sensitivity Analysis – Mediation Model with Severe Forms of NSSI
- Additional Information on Exploratory Analysis Related to Three-Way Interaction.

**Additional Information on Sensitivity Analysis - Mediation Model with Schools that Participated at both Waves**

To examine the robustness of the analysis a sensitivity analysis was conducted on the mediation model between pre-existing vulnerabilities (i.e., prior history of NSSI; internalizing symptoms; poor regulatory emotional self-efficacy) and NSSI at T2 through Covid-19 related stress, including only the schools and classes that participated in both data collection (N = 693). The results of the mediation model with the sample of 693 are consistent with the results of the whole sample. Findings showed that Covid-19 related stress mediates the association between prior history of NSSI (β = 0.065, *SE* = 0.020, *p* = 0.001), internalizing symptoms (β = 0.164, *SE* = 0.028, *p*<0.001), poor regulatory negative emotional self-efficacy (β = 0.104, *SE* = 0.030, *p*<0.001) and the occurrence of NSSI. Specifically, findings showed a positive and significant effect of the previous history of NSSI (β = 0.156, *SE* = 0.043, *p*<0.001), internalizing symptoms (β = 0.391, *SE* = 0.042, *p*<0.001), and poor regulatory negative emotional self-efficacy (β = 0.156, *SE* = 0.043, *p*<0.012) on Covid-19 related stress. Subsequent Covid-19 related stress was positively associated with the occurrence of NSSI (β = 0.420, *SE* = 0.061, *p*<0.001). No significant indirect effects were found on the frequency of non-suicidal self-harm behavior (i.e., continuous variable).

As for the interaction effect between peer perceived support and Covid-19 related stress, a no significant effect was found both on the presence of NSSI at T2 (β = -0.010, *SE* = 0.060, *p* = 0.863) and on the frequency of NSSI at T2 (β = -0.057, *SE* = 0.066, *p* = 0.390). Similar results were also found with respect to the interaction between perceived parental support and Covid-19-related stress on the presence of NSSI at T2 (β = -0.055, *SE* = 0.066, *p* = 0.404) as well as on the frequency of NSSI at T2 (β = 0.018, *SE* = 0.079, *p* = 0.823).

**Additional Information on Sensitivity Analysis – Mediation Model with Severe Forms of NSSI**

The prevalence of NSSI in this sample, although not uncommon (e.g., Cipriano et al., 2017; Lloyd-Richardson et al., 2007; Calvete et al., 2015), was substantially higher than the one that emerged in prior systematic reviews or meta-analyses conducted among normative samples (e.g., Brown & Plener, 2017). A possible reason could be due to adolescents’ engagement in minor/mild NSSI forms like *“self-hitting”* and *“self-biting”* (Whitlock et al., 2014; Kaess et al., 2013), rather than more severe forms, such as *“self-cutting/carving, self-burning, self-scrapping, or inserting object under the skin/nails”* (Kaess et al., 2013; Klonsky & Olino, 2008). Thus, the prevalence of NSSI was also calculated using only the more severe forms of NSSI, removing the items about self-hitting and self-biting, which were the most prevalent in this sample.

Results showed that among adolescents who participated at both time points, 18.50% (instead of 33.1%) reported NSSI at Time 1 and 22% (instead of 34.80%) at T2. Subsequently, sensitivity analyses were conducted to examine whether results replicated when excluding mild forms of NSSI. Overall, results emerged to be highly consistent with the ones from models including all original six items of NSSI. Regarding the occurrence of NSSI, results showed a significant positive effect from prior history of NSSI (β = 0.326, *SE* = 0.053, *p*<0.001). No significant effect of internalizing symptoms (β = 0.069, *SE* = 0.072, *p* = 0.340) and poor regulatory emotion self-efficacy (β = -0.111, *SE* = 0.075, *p* = 0.137) on NSSI at T2 was found. Regarding the frequency of NSSI, no significant effects were found from prior history of NSSI (β = 0.169, *SE* = 0.098, *p* = 0.085), regulatory emotion self-efficacy (β = -0.177, *SE* = 0.145, *p* = 0.223) as well as internalizing symptoms (β = 0.119, *SE* = 0.154, *p* = 0.440) on the frequency of NSSI at T2. Notably, this latter result together with the effect of poor regulatory self-efficacy on the occurrence of NSSI is the only one that somewhat diverged from the primary study analyses, despite the highly comparable effect size.

Consistent with the main findings results also revealed indirect effects from the prior history of NSSI (β = 0.038, *SE* = 0.015, *p* = 0.011), internalizing symptoms (β = 0.132, *SE* = 0.031, *p*<0.001), and poor regulatory negative emotional self-efficacy (β = 0.076, *SE* = 0.027, *p* = 0.004) on the occurrence of NSSI at T2 via higher levels of Covid-19 related stress. No significant indirect effects were found on the frequency of NSSI. As for the interaction effects, neither peer nor parental support moderated the associations between Covid-19 related stress and the occurrence of NSSI at T2 (respectively β = 0.006, *SE* = 0.064, *p* = 0.928 and β = 0.068, *SE* = 0.066, *p* = 0.302) as well as the frequency of NSSI at T2 (respectively β = -0.098, *SE* = 0.078, *p* = 0.211 and β = - 0.104, *SE* = 0.095, *p* = 0.273).

**Additional Information on Exploratory Analysis Related to Three-Way Interaction**

A three-way interaction between social support at both waves and Covid-19 related stress was conducted to explore the possible changes in social support over time. Specifically, the interactions were tested between (1) social support (e.g., peer and parental support) at T1 and Covid-19 related stress; (2) social support at T2 and Covid-19 related stress; (3) social support at T1, social support at T2 and Covid-19 related stress, and finally (4) social support at T1 and social support at T2. Findings showed no significant effect of the different interactions considered both on the presence and frequency of NSSI at T2. Below the results for peer and parental support are presented.

*Peer Support*

Findings showed not significant effect for the interaction between Covid-19 related stress and peer support at T1 both on the presence of NSSI at T2 (β = -0.107, *SE* = 0.086, *p* = 0.214) and on the frequency of NSSI at T2 (β = -0.106, *SE* = 0.131, *p* = 0.419).

No significant results were found for the interaction between Covid-19 related stress and peer support at T2 both on the presence of NSSI at T2 (β = 0.028, *SE* = 0.064, *p* = 0.663) and on the frequency of NSSI at T2 (β = -0.045, *SE* = 0.076, *p* = 0.560).

As the interaction between Covid-19 related stress, peer support at T1 and peer support at T2, a not significant effect was found both on the occurrence of NSSI at T2 (β = 0.119, *SE* = 0.139, *p* = 0.392) and on the frequency of NSSI at T2 (β = -0.056, *SE* = 0.130, *p* = 0.668).

Finally, no significant results were found for the interaction between peer support at T1 and peer support at T2 both on the occurrence of NSSI at T2 (β = 0.025, *SE* = 0.091, *p* = 0.782) and on the frequency of NSSI at T2 (β = -0.054, *SE* = 0.119, *p* = 0.646).

*Parental Support*

Similar results were also found for the perceived parental support. Specifically, a no significant effect of the interaction between Covid-19 related stress and parental support at T1 was found both on the presence of NSSI at T2 (β = -0.008, SE = 0.103, p = 0.938) and on the frequency of NSSI at T2 (β = -0.119, *SE* = 0.153, *p* = 0.436).

As the interaction between Covid-19 related stress and parental support at T2, a no significant effect was found both on the presence of NSSI at T2 (β = -0.057, *SE* = 0.076, *p* = 0.455) and on the frequency of NSSI at T2 (β = 0.051, *SE* = 0.094, *p* = 0.589).

No significant effects were found for the interaction between Covid-19 related stress, parental support at T1 and parental support at T2 both on the occurrence of NSSI at T2 (β = 0.092, *SE* = 0.166, *p* = 0.579) and on the frequency of NSSI at T2 (β = -0.054, *SE* = 0.166, *p* = 0.747).

Finally, similar results were found for the interaction between parental support at T1 and parental support at T2 both on the occurrence of NSSI at T2 (β = -0.075, *SE* = 0.116, *p* = 0.518) and on the frequency of NSSI at T2 (β = 0.035, *SE* = 0.136, *p* = 0.795).
